# Bromido(1,4,7,10,13-penta­aza­cyclo­hexa­deca­ne)cobalt(III) dibromide dihydrate

**DOI:** 10.1107/S1600536813004947

**Published:** 2013-03-02

**Authors:** Tsutomu Kurisaki, Makoto Hamano, Hisanobu Wakita

**Affiliations:** a Department of Chemistry, Faculty of Science, Fukuoka University, Nanakuma, Jonan-ku, Fukuoka 814-0180, Japan

## Abstract

The title salt, [CoBr(C_11_H_27_N_5_)]Br_2_·2H_2_O, contains a complex cation with mirror symmetry and two Br^−^ counter-anions that are likewise located on the mirror plane. The central Co^III^ atom of the complex cation has one Br^−^ ion in an axial position, one N atom of the penta­dentate macrocyclic ligand in the other axial position and four N atoms of the ligand in equatorial positions, defining a distorted octa­hedral coordination geometry. The macrocyclic ligand is coordinated to the Co^III^ atom within a 5, 6, 5 arrangement of chelate rings in the equatorial plane of the four N atoms. Due to symmetry, the configuration of the chiral N atoms is 1*RS*, 4*SR*, 10*RS*, 13*SR*. In the crystal, N—H⋯Br, O—H⋯Br and N—H⋯O hydrogen bonds between the complex cation, anions and lattice water mol­ecules generate a three-dimensional network.

## Related literature

For background to metal complexes with aza­macrocycles, see: Mewis & Archida (2010 ▶). For related structures, see: Curtis *et al.* (1987*a* ▶,*b* ▶); Eigenbrot *et al.* (1988 ▶); Tahirov *et al.* (1993 ▶); Bombieri *et al.* (1982 ▶). For the synthesis of the macrocyclic ligand, see: Richman & Atkins (1974 ▶).
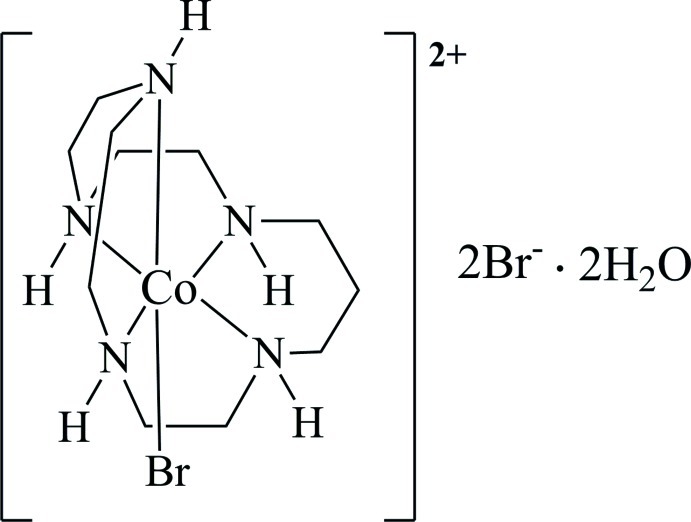


## Experimental

### 

#### Crystal data

[CoBr(C_11_H_27_N_5_)]Br_2_·2H_2_O
*M* *_r_* = 564.07
Orthorhombic, 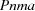

*a* = 13.139 (3) Å
*b* = 9.6674 (18) Å
*c* = 15.393 (3) Å
*V* = 1955.2 (7) Å^3^
*Z* = 4
Mo *K*α radiation
μ = 7.02 mm^−1^
*T* = 296 K
0.40 × 0.22 × 0.14 mm

#### Data collection

Rigaku Saturn724+ diffractometer
Absorption correction: numerical (*NUMABS*; Rigaku, 1999 ▶) *T*_min_ = 0.189, *T*_max_ = 0.501
28744 measured reflections
2370 independent reflections
1964 reflections with *I* > 2σ(*I*)
*R*_int_ = 0.075

#### Refinement

*R*[*F* ^2^ > 2σ(*F* ^2^)] = 0.031
*wR*(*F* ^2^) = 0.076
*S* = 1.02
2370 reflections
109 parameters
4 restraints
H-atom parameters constrained
Δρ_max_ = 0.89 e Å^−3^
Δρ_min_ = −0.88 e Å^−3^

### 

Data collection: *CrystalClear* (Rigaku, 2008 ▶); cell refinement: *CrystalClear*; data reduction: *CrystalClear*; program(s) used to solve structure: *SIR92* (Altomare *et al.*, 1993 ▶); program(s) used to refine structure: *SHELXL97* (Sheldrick, 2008 ▶); molecular graphics: *ORTEP-3 for Windows* (Farrugia, 2012 ▶); software used to prepare material for publication: *WinGX* (Farrugia, 2012 ▶).

## Supplementary Material

Crystal structure: contains datablock(s) I, global. DOI: 10.1107/S1600536813004947/wm2723sup1.cif

Structure factors: contains datablock(s) I. DOI: 10.1107/S1600536813004947/wm2723Isup3.hkl

Supplementary material file. DOI: 10.1107/S1600536813004947/wm2723Isup4.cdx

Additional supplementary materials:  crystallographic information; 3D view; checkCIF report

## Figures and Tables

**Table 1:** Hydrogen-bond geometry (Å, °)

*D*—H⋯*A*	*D*—H	H⋯*A*	*D*⋯*A*	*D*—H⋯*A*
N1—H1⋯Br2	0.91	2.5	3.303 (3)	147
N2—H2⋯Br3	0.91	2.57	3.415 (2)	155
N3—H3⋯O*W* ^i^	0.91	2.24	3.060 (4)	150
O*W*—H*W*2⋯Br2	0.87	2.64	3.491 (3)	168
O*W*—H*W*1⋯Br3^ii^	0.82	2.67	3.489 (3)	170

Symmetry codes: (i) 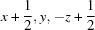; (ii) 
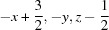
.

**Fig. 1.**
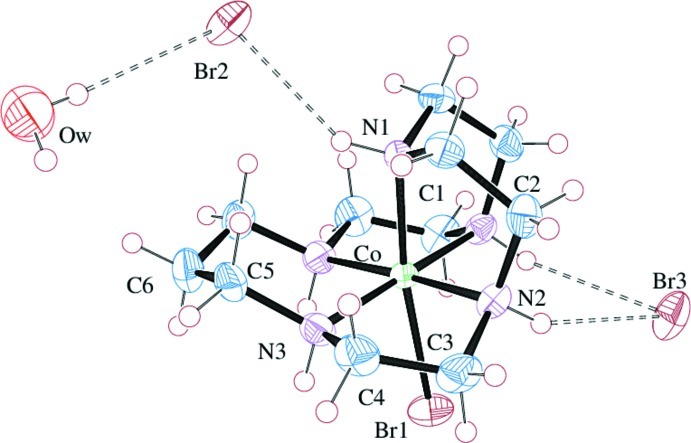
The molecular structure of the title compound, with 50% probability displacement ellipsoids and the atom-numbering scheme.

**Fig. 2.**
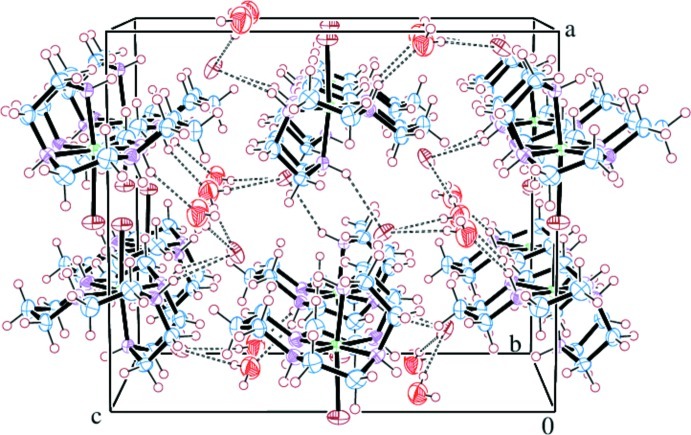
Crystal Structure of the title compound with view along the b axis. Intermolecular hydrogen bonding is shown as dashed lines.

## supplementary crystallographic information

### Comment

Azamacrocycles are popular ligands for the preparation of metal complexes because of their stability and defined geometry. These ligands often possess enough conformational freedom for their intended functionalities (Mewis & Archida, 2010). The coordination of pentaaza macrocycles to the cobalt(III) ion can result in a number of isomeric forms. The complexes formed between cobalt(III) and a series of eight pentaaza macrocycles with ring sizes varying from 15- to 20-membered rings have been investigated (Curtis *et al.*, 1987**a*,b*). These cobalt(III) complexes may exist as three diastereoisomers, *i.e.**meso*-*syn*, *meso-anti*, and the racemic isomer. For the cobalt(III) complex of 1,4,7,11,14-pentaazacycloheptadecane ([17]aneN_5_) it has been reported that two isomeric forms could be isolated. The crystal structures of these two isomers, [CoBr([17]aneN_5_)][ZnBr_4_] (Eigenbrot *et al.*, 1988) and [CoCl([17]aneN_5_)]Cl(ClO_4_) (Tahirov *et al.*, 1993), have been determined as the racemic and the *meso-anti* isomer, respectively. Furthermore, the cobalt(III) complex of 1,4,7,11,14-pentaazacyclohexadecane ([16]aneN_5_), [CoCl([16]aneN_5_)](ClO_4_)_2_, crystallized as the *meso*-*syn* isomer (Bombieri *et al.*, 1982).

In the title complex, [CoBr(C_11_H_27_N_5_)]Br_2_^.^2H_2_O, the Co^III^ atom is surrounded by one Br^-^ anion and N atoms of the macrocyclic ligand to form a distorted octahedral environment (Fig.1). The Co—N(axial) bond in the complex is longer than the Co—N(equatorial) bonds, presumably caused by the *trans* effect of the Br atom. The average Co—N(equatorial) distance of 1.967 Å is shorter than that in cobalt(III) complexes of 1,4,7,11,14-pentaazacycloheptadecane (Eigenbrot *et al.*, 1988) and 1,4,7,11,15-pentaazacyclooctadecane (Curtis *et al.*, 1987*a*). The macrocyclic ligand adopts a stable conformation with the one six-membered chelate ring in chair form and four five-membered chelate rings in *gauche* forms. The macrocyclic ligand is coordinated in a configuration with five-, six-, and five-membered chelate rings in the equatorial plane. The deviation of the Co^III^ atom from the equatorial plane is 0.03 A. The N3 and N3* atoms have opposite chirality giving the *meso*-*syn* diastereoisomer. The macrocyclic ligand coordinates in the *meso*-*syn* configuration with hydrogen atoms on N2, N2*, N3, and N3* on the same side of the equatorial plane relative to the axially coordinating bromide anion. Due to mirror symmetry of the entire complec cation, the configurations of the four chiral amine N atoms are 1RS, 4SR, 10RS, and 13SR. Hydrogen bonds between N atoms of the macrocyclic ligand, water molecules and bromide counter anions exists (Fig. 2; Table 1), stabilizing the crystal packing within a three-dimensional network.

### Experimental

The ligand 1,4,7,10,13-pentaazacyclohexadecane pentahydrobromide was prepared according to the literature method (Richman & Atkins, 1974). The ligand (1.26 g, 2 mmol) was dissolved in water and treated with freshly prepared Na_3_[Co(CO_3_)_3_]^.^3H_2_O (0.72 g, 2 mmol). The mixture was refluxed for 1 h and filtered. To the filtrate was added NH_4_Br in excess and the solution allowed to stand for several days whereupon dark violet crystals of the title compound were formed.

### Refinement

All H atoms attached to C and N atoms were placed geometrically (C—H = 0.97 and N—H = 0.91 Å) and were refined as riding with *U*_iso_(H) = 1.2*U*_eq_(C,*N*). The water H atoms were located in difference Fourier maps and were refined initially with restrains O—H = 0.85 (2) Å. In the last cycles of refinement, they were eventually refined as riding, with *U*_iso_(H) = 1.5*U*_eq_(O).

### Crystal data

**Table:**

[CoBr(C_11_H_27_N_5_)]Br_2_·2H_2_O	*F*(000) = 1120
*M**_r_* = 564.07	*D*_x_ = 1.916 Mg m^−^^3^
Orthorhombic, *P**n**m**a*	Mo *K*α radiation, λ = 0.71073 Å
Hall symbol: -P 2ac 2n	Cell parameters from 4654 reflections
*a* = 13.139 (3) Å	θ = 3.1–27.5°
*b* = 9.6674 (18) Å	µ = 7.02 mm^−^^1^
*c* = 15.393 (3) Å	*T* = 296 K
*V* = 1955.2 (7) Å^3^	Prism, dark violet
*Z* = 4	0.40 × 0.22 × 0.14 mm

### Data collection

**Table:**

Rigaku Saturn724+ diffractometer	2370 independent reflections
Radiation source: rotating anode	1964 reflections with *I* > 2σ(*I*)
Graphite monochromator	*R*_int_ = 0.075
Detector resolution: 28.5714 pixels mm^-1^	θ_max_ = 27.5°, θ_min_ = 3.1°
dtprofit.ref scans	*h* = −17→17
Absorption correction: numerical (*NUMABS*; Rigaku, 1999)	*k* = −12→12
*T*_min_ = 0.189, *T*_max_ = 0.501	*l* = −19→19
28744 measured reflections	

### Refinement

**Table:**

Refinement on *F*^2^	Primary atom site location: structure-invariant direct methods
Least-squares matrix: full	Secondary atom site location: difference Fourier map
*R*[*F*^2^ > 2σ(*F*^2^)] = 0.031	Hydrogen site location: inferred from neighbouring sites
*wR*(*F*^2^) = 0.076	H-atom parameters constrained
*S* = 1.02	*w* = 1/[σ^2^(*F*_o_^2^) + (0.0392*P*)^2^] where *P* = (*F*_o_^2^ + 2*F*_c_^2^)/3
2370 reflections	(Δ/σ)_max_ = 0.001
109 parameters	Δρ_max_ = 0.89 e Å^−^^3^
4 restraints	Δρ_min_ = −0.88 e Å^−^^3^

### Special details

**Table:**

Geometry. All e.s.d.'s (except the e.s.d. in the dihedral angle between two l.s. planes) are estimated using the full covariance matrix. The cell e.s.d.'s are taken into account individually in the estimation of e.s.d.'s in distances, angles and torsion angles; correlations between e.s.d.'s in cell parameters are only used when they are defined by crystal symmetry. An approximate (isotropic) treatment of cell e.s.d.'s is used for estimating e.s.d.'s involving l.s. planes.
Refinement. Refinement of *F*^2^ against ALL reflections. The weighted *R*-factor *wR* and goodness of fit *S* are based on *F*^2^, conventional *R*-factors *R* are based on *F*, with *F* set to zero for negative *F*^2^. The threshold expression of *F*^2^ > σ(*F*^2^) is used only for calculating *R*-factors(gt) *etc*. and is not relevant to the choice of reflections for refinement. *R*-factors based on *F*^2^ are statistically about twice as large as those based on *F*, and *R*- factors based on ALL data will be even larger.

### Fractional atomic coordinates and isotropic or equivalent isotropic displacement parameters (Å^2^)

**Table:**

	*x*	*y*	*z*	*U*_iso_*/*U*_eq_	
Br1	0.97038 (3)	0.25	0.51348 (3)	0.04287 (14)	
Br2	0.44795 (3)	0.25	0.38291 (3)	0.04299 (14)	
Br3	0.89341 (4)	0.25	0.77417 (3)	0.04833 (15)	
Co	0.78735 (4)	0.25	0.50953 (3)	0.02053 (13)	
OW	0.4692 (2)	0.0362 (3)	0.20106 (17)	0.0743 (9)	
HW1	0.5085	−0.0263	0.2156	0.111*	
HW2	0.4701	0.0986	0.2416	0.111*	
N1	0.6374 (2)	0.25	0.5240 (2)	0.0227 (7)	
H1	0.6083	0.25	0.4703	0.027*	
N2	0.78844 (16)	0.0990 (2)	0.59473 (15)	0.0272 (5)	
H2	0.8333	0.1219	0.6374	0.033*	
N3	0.79176 (17)	0.1035 (2)	0.42054 (15)	0.0298 (5)	
H3	0.8556	0.1076	0.3977	0.036*	
C1	0.60554 (19)	0.1226 (3)	0.57002 (19)	0.0309 (6)	
H1A	0.5957	0.0482	0.5286	0.037*	
H1B	0.5416	0.1385	0.5998	0.037*	
C2	0.6869 (2)	0.0825 (3)	0.63512 (19)	0.0344 (7)	
H2A	0.6818	0.1408	0.6862	0.041*	
H2B	0.6773	−0.0129	0.653	0.041*	
C3	0.8268 (2)	−0.0279 (3)	0.5511 (2)	0.0395 (7)	
H3A	0.8063	−0.109	0.5838	0.047*	
H3B	0.9006	−0.0258	0.5488	0.047*	
C4	0.7838 (2)	−0.0351 (3)	0.4602 (2)	0.0396 (7)	
H4A	0.8217	−0.1018	0.4259	0.047*	
H4B	0.7132	−0.0641	0.4621	0.047*	
C5	0.7214 (2)	0.1176 (3)	0.34490 (19)	0.0369 (7)	
H5A	0.6517	0.1154	0.3656	0.044*	
H5B	0.7309	0.0392	0.3064	0.044*	
C6	0.7384 (4)	0.25	0.2943 (3)	0.0452 (12)	
H6A	0.6932	0.25	0.2445	0.054*	
H6B	0.8076	0.25	0.2724	0.054*	

### Atomic displacement parameters (Å^2^)

**Table:**

	*U*^11^	*U*^22^	*U*^33^	*U*^12^	*U*^13^	*U*^23^
Br1	0.0225 (2)	0.0483 (3)	0.0578 (3)	0	0.00093 (19)	0
Br2	0.0392 (3)	0.0353 (3)	0.0546 (3)	0	−0.0198 (2)	0
Br3	0.0605 (3)	0.0443 (3)	0.0402 (3)	0	−0.0220 (2)	0
Co	0.0186 (3)	0.0205 (3)	0.0225 (3)	0	0.00025 (19)	0
OW	0.082 (2)	0.087 (2)	0.0533 (17)	0.0336 (16)	−0.0023 (14)	0.0002 (15)
N1	0.0247 (16)	0.0235 (16)	0.0198 (16)	0	0.0005 (13)	0
N2	0.0270 (12)	0.0243 (12)	0.0301 (13)	−0.0013 (9)	−0.0066 (10)	0.0031 (10)
N3	0.0284 (12)	0.0313 (13)	0.0297 (13)	0.0026 (10)	0.0031 (10)	−0.0055 (11)
C1	0.0261 (14)	0.0318 (16)	0.0349 (16)	−0.0057 (11)	0.0035 (12)	0.0017 (12)
C2	0.0421 (17)	0.0318 (16)	0.0293 (16)	−0.0035 (13)	0.0006 (13)	0.0094 (13)
C3	0.0450 (18)	0.0248 (15)	0.049 (2)	0.0108 (13)	−0.0048 (15)	0.0010 (14)
C4	0.0480 (19)	0.0256 (16)	0.0451 (18)	0.0056 (13)	0.0024 (16)	−0.0090 (14)
C5	0.0392 (17)	0.0436 (18)	0.0280 (15)	0.0022 (14)	0.0001 (13)	−0.0145 (14)
C6	0.049 (3)	0.062 (3)	0.025 (2)	0	0.000 (2)	0

### Geometric parameters (Å, º)

**Table:**

Br1—Co	2.4056 (8)	C1—C2	1.516 (4)
Co—N2^i^	1.962 (2)	C1—H1A	0.97
Co—N2	1.962 (2)	C1—H1B	0.97
Co—N3	1.971 (2)	C2—H2A	0.97
Co—N3^i^	1.971 (2)	C2—H2B	0.97
Co—N1	1.982 (3)	C3—C4	1.512 (4)
OW—HW1	0.8249	C3—H3A	0.97
OW—HW2	0.8682	C3—H3B	0.97
N1—C1^i^	1.482 (3)	C4—H4A	0.97
N1—C1	1.482 (3)	C4—H4B	0.97
N1—H1	0.91	C5—C6	1.516 (4)
N2—C2	1.481 (3)	C5—H5A	0.97
N2—C3	1.486 (3)	C5—H5B	0.97
N2—H2	0.91	C6—C5^i^	1.516 (4)
N3—C4	1.476 (4)	C6—H6A	0.97
N3—C5	1.493 (4)	C6—H6B	0.97
N3—H3	0.91		
			
N2^i^—Co—N2	96.11 (14)	N1—C1—H1A	109.8
N2^i^—Co—N3	177.03 (10)	C2—C1—H1A	109.8
N2—Co—N3	85.97 (10)	N1—C1—H1B	109.8
N2^i^—Co—N3^i^	85.97 (10)	C2—C1—H1B	109.8
N2—Co—N3^i^	177.03 (10)	H1A—C1—H1B	108.3
N3—Co—N3^i^	91.87 (14)	N2—C2—C1	109.3 (2)
N2^i^—Co—N1	86.12 (9)	N2—C2—H2A	109.8
N2—Co—N1	86.12 (9)	C1—C2—H2A	109.8
N3—Co—N1	96.15 (9)	N2—C2—H2B	109.8
N3^i^—Co—N1	96.15 (9)	C1—C2—H2B	109.8
N2^i^—Co—Br1	88.61 (6)	H2A—C2—H2B	108.3
N2—Co—Br1	88.61 (6)	N2—C3—C4	109.3 (2)
N3—Co—Br1	89.32 (7)	N2—C3—H3A	109.8
N3^i^—Co—Br1	89.32 (7)	C4—C3—H3A	109.8
N1—Co—Br1	172.11 (9)	N2—C3—H3B	109.8
HW1—OW—HW2	107.8	C4—C3—H3B	109.8
C1^i^—N1—C1	112.5 (3)	H3A—C3—H3B	108.3
C1^i^—N1—Co	109.54 (16)	N3—C4—C3	108.3 (2)
C1—N1—Co	109.54 (16)	N3—C4—H4A	110
C1^i^—N1—H1	108.4	C3—C4—H4A	110
C1—N1—H1	108.4	N3—C4—H4B	110
Co—N1—H1	108.4	C3—C4—H4B	110
C2—N2—C3	113.9 (2)	H4A—C4—H4B	108.4
C2—N2—Co	110.77 (16)	N3—C5—C6	112.8 (3)
C3—N2—Co	108.34 (18)	N3—C5—H5A	109
C2—N2—H2	107.9	C6—C5—H5A	109
C3—N2—H2	107.9	N3—C5—H5B	109
Co—N2—H2	107.9	C6—C5—H5B	109
C4—N3—C5	111.2 (2)	H5A—C5—H5B	107.8
C4—N3—Co	111.29 (18)	C5—C6—C5^i^	115.3 (4)
C5—N3—Co	117.28 (17)	C5—C6—H6A	108.4
C4—N3—H3	105.3	C5^i^—C6—H6A	108.4
C5—N3—H3	105.3	C5—C6—H6B	108.4
Co—N3—H3	105.3	C5^i^—C6—H6B	108.4
N1—C1—C2	109.2 (2)	H6A—C6—H6B	107.5

Symmetry code: (i) *x*, −*y*+1/2, *z*.

### Hydrogen-bond geometry (Å, º)

**Table:**

*D*—H···*A*	*D*—H	H···*A*	*D*···*A*	*D*—H···*A*
N1—H1···Br2	0.91	2.5	3.303 (3)	147
N2—H2···Br3	0.91	2.57	3.415 (2)	155
N3—H3···O*W*^ii^	0.91	2.24	3.060 (4)	150
O*W*—H*W*2···Br2	0.87	2.64	3.491 (3)	168
O*W*—H*W*1···Br3^iii^	0.82	2.67	3.489 (3)	170

Symmetry codes: (ii) *x*+1/2, *y*, −*z*+1/2; (iii) −*x*+3/2, −*y*, *z*−1/2.
